# Emerging Genotype IV Japanese Encephalitis Virus Outbreak in New South Wales, Australia

**DOI:** 10.3390/v14091853

**Published:** 2022-08-24

**Authors:** Annaleise R. Howard-Jones, David Pham, Neisha Jeoffreys, John-Sebastian Eden, Linda Hueston, Alison M. Kesson, Vanathi Nagendra, Harsha Samarasekara, Peter Newton, Sharon C.-A. Chen, Matthew V. O’Sullivan, Susan Maddocks, Dominic E. Dwyer, Jen Kok

**Affiliations:** 1 Centre for Infectious Diseases & Microbiology Laboratory Services, New South Wales Health Pathology-Institute of Clinical Pathology and Medical Research, Westmead Hospital, Westmead, NSW 2145, Australia; 2 Faculty of Medicine and Health, University of Sydney, Camperdown, NSW 2050, Australia; 3 Sydney Infectious Diseases Institute, The University of Sydney, Westmead, NSW 2145, Australia; 4 Centre for Virus Research, Westmead Institute for Medical Research, Westmead, NSW 2145, Australia; 5 Department of Infectious Disease & Microbiology, The Children’s Hospital at Westmead, Westmead, NSW 2145, Australia; 6 Department of Microbiology and Infectious Diseases, NSW Health Pathology-Liverpool, Sydney, NSW 2170, Australia; 7 Department of Microbiology, Orange Base Hospital, Orange, NSW 2800, Australia; 8 Department of Microbiology, New South Wales Health Pathology-Nepean, Nepean Hospital, Kingswood, NSW 2747, Australia; 9 Microbiology, NSW Health Pathology-Wollongong, Wollongong Hospital, Wollongong, NSW 2500, Australia

**Keywords:** Japanese encephalitis, flavivirus, outbreak, serology, nucleic acid testing, molecular diagnostics, metagenomics

## Abstract

The detection of a new and unexpected Japanese encephalitis virus (JEV) outbreak in March 2022 in Australia, where JEV is not endemic, demanded the rapid development of a robust diagnostic framework to facilitate the testing of suspected patients across the state of New South Wales (NSW). This nascent but comprehensive JEV diagnostic service encompassed serological, molecular and metagenomics testing within a centralised reference laboratory. Over the first three months of the outbreak (4 March 2022 to 31 May 2022), 1,061 prospective samples were received from 878 NSW residents for JEV testing. Twelve confirmed cases of Japanese encephalitis (JE) were identified, including ten cases diagnosed by serology alone, one case by metagenomic next generation sequencing and real-time polymerase chain reaction (RT-PCR) of brain tissue and serology, and one case by RT-PCR of cerebrospinal fluid, providing an incidence of JE over this period of 0.15/100,000 persons in NSW. As encephalitis manifests in <1% of cases of JEV infection, the population-wide prevalence of JEV infection is likely to be substantially higher. Close collaboration with referring laboratories and clinicians was pivotal to establishing successful JEV case ascertainment for this new outbreak. Sustained and coordinated animal, human and environmental surveillance within a OneHealth framework is critical to monitor the evolution of the current outbreak, understand its origins and optimise preparedness for future JEV and arbovirus outbreaks.

## 1. Introduction

**Figure 1:**
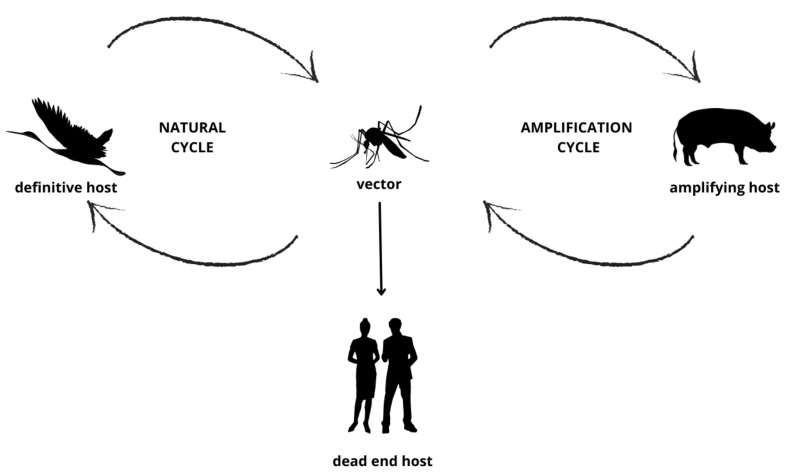
Schematic of Japanese encephalitis virus (JEV) circulation amongst mosquitoes (vectors), wading birds (definitive hosts), pigs (amplifying hosts) and humans (dead end hosts). Of note, humans and other ‘dead end hosts’, for example horses, llamas, dogs, goats, do not develop high-level viraemia and hence carry a minimal risk of transmitting the virus to mosquitoes and hence perpetuating the JEV lifecycle.

Japanese encephalitis virus (JEV) is a mosquito-borne arbovirus of the family Flaviviridae, and the cause of human Japanese encephalitis (JE) (Figure 1), a condition with potentially severe and long-lasting clinical sequelae [1]. JEV is closely related to other encephalitic flaviviruses endemic within Australia such as Murray Valley Encephalitis Virus (MVEV) and the Kunjin variant of West Nile Virus (WNV_KUNV_) [2], as well as other imported flaviviruses, including dengue, yellow fever and Zika viruses [1].

Although well-established throughout South-East Asia and the Pacific, widespread transmission of JEV has not previously been described in Australia [3]. Prior to 2022, cases were largely confined to returned travellers from endemic regions, with only five cases of locally transmitted infection in the Torres Strait and Cape York in Far North Queensland from 1995–1998 [4]. In 2021, a sentinel case of JEV infection (belonging to genotype IV) was detected in the Tiwi Islands (located 80 kilometres north of Darwin in the Northern Territory) of Australia [5], with no further clinical cases reported in the subsequent months.

The current JEV outbreak was first detected in swine within commercial piggeries on 26 February 2022 [6], and subsequently in humans, mosquito pools and sentinel chickens. The outbreak was declared a Communicable Disease Incident of National Significance (CDINS) by the Australian Acting Chief Medical Officer on 4 March 2022 [7]. Based on phylogenetic analyses of the envelope gene, the virus was identified as genotype IV, the least common of the five JEV genotypes (I to V) worldwide [8].

Although most JEV infections are asymptomatic or present with a mild non-specific febrile illness, the encephalitic manifestation (JE) may lead to high morbidity and mortality [9]. The virus is neurotropic with a predilection for the thalamus. Key clinical features of JE include Parkinsonian-like signs, seizures (particularly in children) and altered level of consciousness; rarely, Guillain–Barré syndrome and an acute flaccid paralysis have been described [1]. Mortality rates in this form of encephalitis are high, averaging 18% in one meta-analysis (95% CI 14–21%) [10], and long-term neurological sequelae, including brain stem dysfunction, ‘locked-in syndrome’ and seizure disorders, are reported in over 40% of survivors [10,11,12]. In endemic areas, children are commonly affected as evidenced by seroprevalence data [1], though this pattern is unlikely to be replicated in largely seronegative populations such as in Australia.

This study presents a cross-sectional analysis of JEV diagnoses during the emergent phase of the outbreak in New South Wales (NSW), Australia. We present data on JEV case detections across NSW using a multi-modal approach [8] and discuss challenges in confirming JEV diagnosis in a population where JEV is not endemic, but other flaviviruses are circulating.

## 2. Materials and Methods

With the declaration of JEV as a CDINS, specimens from persons with suspected JEV infection across NSW were referred to the NSW Health Pathology-Institute of Clinical Pathology and Medical Research (NSWHP-ICPMR) laboratory for testing. Information was distributed to all NSW public and private laboratories to advise microbiologists and laboratory staff regarding the range of JEV-associated clinical syndromes, acceptable sample types and available tests. Referral for prospective testing was at the discretion of the clinician in consultation with the NSWHP-ICPMR microbiologist on-call; samples included in this cross-sectional analysis were received from 4 March 2022 to 31 May 2022. Testing modalities are outlined in Table 1. 

In addition, NSW laboratories were invited to submit stored cerebrospinal fluid (CSF) specimens collected from persons with clinical encephalitis or CSF pleocytosis (white cell count ≥5 cells/microL) from 1 October 2021 to 3 March 2022, where a pathogen had not been identified by routine testing (bacterial culture and nucleic acid amplification testing [NAAT] for routine pathogens causing encephalitis and/or meningitis). 

Due to clinical and laboratory constraints, JEV testing was offered only for clinical cases of encephalitis. Deidentified demographic data (comprising age, sex and postcode of residence) were collected from each laboratory’s information system and analysed in aggregate.

**Ethics and patient consent:** Clinical specimens were received and processed at the NSWHP-ICPMR laboratory for standard-of-care diagnostic purposes. A non-research determination was granted by Health Protection NSW as a designated communicable disease control activity, encompassing prospective and retrospective samples. Samples were deidentified prior to analysis, and data were analysed in aggregate only.

**Serological assays:** JEV-specific serology was performed on both serum and CSF samples submitted during the study timeframe, and the collection of acute and convalescent samples was recommended (the latter collected two to four weeks after illness onset). For sera, a defined epitope blocking (DEB) enzyme-linked immunosorbent assay (ELISA) was used to assess for JEV-specific total antibody according to established procedures [13,14], using an in-house viral antigen preparation and horseradish peroxidase-labelled monoclonal antibody (JE/989) [13]. All positive serum samples by DEB ELISA were also tested for JEV-specific IgM and IgG by immunofluorescence (IF) at 1/10 dilution. JEV-specific IgM IF testing was also performed on undiluted and 1/10 diluted CSF samples. For IF testing, slides were prepared using confluent Vero E6 cell monolayers infected with the Nakayama JEV strain. Fluorescein-labelled antihuman IgG, IgA and IgM were used for visualisation with examination by fluorescence microscopy. All positive results on IF were retested in a titration series (1/10 to 1/1280) to determine an endpoint titre. Testing of IgM and/or IgG specific to other flaviviruses was performed in parallel on all positive samples to exclude potential false positive JEV results due to cross-reactivity. All such samples were tested for WNV_KUNV_- and MVEV-specific antibodies; dengue, Zika and/or yellow fever virus testing was added where a specific travel history was elicited.

**Nucleic acid amplification testing:** CSF, whole blood, urine, and brain tissue were accepted for JEV-specific real-time polymerase chain reaction (RT-PCR) testing using published methods [15], with one minor modification to the reverse primer (utilising Y rather than T in the terminal nucleotide) to improve sensitivity. The assay was multiplexed to include the human β-globin (HBG) gene target [16] as an internal control for sample adequacy and nucleic acid extraction. Nucleic acid was extracted from clinical samples using either the High Pure PCR Template Preparation Kit (Roche Diagnostics GmbH, Mannheim, Germany) for whole blood and brain tissue samples, the NucliSENS easyMAG total nucleic acid extraction system (bioMérieux, Marcy-l’Étoile, France) for CSF, or the MagnaPure MP96 with Small Volume Protocol (Roche Diagnostics) for urine samples. Template nucleic acid (5 µL) was then combined with 400 nM each of Universal JEV forward and reverse primers (5′GCCACCCAGGAGGTCCTT3′; 5′CCCCAAAACCGCAGGAAY3′); 200 nM Universal JEV-MGB-Probe (6FAM-CAAGAGGTG/ZEN/GACGGCC-IABkFQ); 200 nM each of HBG forward and reverse primers (5′GAAGAGCCAAGGACAGGTAC3′; 5′CACCAACTTCATCCACGTTCAC3′); 100 nM HBG probe (TxRd-TCAAACAGACACCATGGTGCACCTG-BHQ2); and 1× AgPath-ID One-Step RT-PCR Reagents (ThermoFisher, Waltham, MA, USA) to provide a final volume of 25 µL. The reaction mix was amplified and detected using the LightCycler 480 (Roche Diagnostics) instrument with the following cycling conditions: (45 °C × 10 min; 95 °C × 10 min) × 1 cycle; (95 °C × 15 sec; 60 °C × 45 sec) × 45 cycles. 

**Metagenomic next generation sequencing (mNGS):** Metagenomics testing using a rapid RNA-based mNGS protocol was offered on a case-by-case basis for patients where clinical suspicion for JE was high but prior testing had not revealed a pathogen [17]. Double-stranded cDNA was prepared from extracted RNA using Invitrogen SuperScript IV VILO Master Mix with ezDNase (ThermoFisher) and Sequenase DNA Polymerase v2 (Applied Biosystems, ThermoFisher). NGS was performed using an Illumina iSeq with the Nextera XT DNA library preparation kit (Illumina, San Diego, CA, USA). For analysis, reads were directly mapped to the JEV genotype IV reference genome or assembled de novo with MEGAHIT [18] before identifying sequences by comparison to NCBI GenBank references using BLAST [19]. 

**Case definitions and prevalence calculations:** Case confirmation of JE was based on nationally published diagnostic criteria [20] (Table 1). For the purposes of this study, a confirmed case of JE required laboratory definitive evidence only—the isolation of JEV by culture; the detection of JEV by NAAT; JEV-specific IgG seroconversion or a significant increase in antibody levels in the absence of recent JEV vaccination; or the detection of JEV-specific IgM in CSF in the absence of IgM to MVEV, WNV_KUNV_ and dengue viruses. A probable case of JE was defined in this study as a case with suggestive laboratory evidence that did not meet criteria for a confirmed case, such as a positive JEV-specific IgM without serological testing for other flaviviruses. Given short-lived and low-level viraemia of JEV in humans, viral culture was not used as a diagnostic tool for acute infection in our laboratory. 

Population estimates were based on the 2016 Census data for NSW from the Australian Bureau of Statistics [21]. Statistical analyses were performed using Microsoft Excel (Microsoft 365, version 2108, Microsoft Corporation, Redmond, US) and R (version 4.2.0, R Foundation for Statistical Computing, Vienna, Austria).

## 3. Results

Over the first three months of the JEV outbreak in NSW, 1061 samples from 878 patients were tested between 4 March 2022 and 31 May 2022 as part of routine contemporaneous diagnostics. The median age of patients tested was 44.3 years (interquartile range (IQR) 29.6 to 58.5 years; range 15 days to 89.5 years) with a 1.13:1 male:female ratio. 

Serological testing was performed on 700 sera from 663 patients over this period (Table 2). JEV-specific IgM was detected in sera from 13 patients, of whom 11 fulfilled criteria for confirmed JEV infection (Table 3). In two cases, the raised JEV-specific IgM was attributed to an alternative diagnosis, namely, Zika virus infection in a returned traveller, and non-specific immunoglobulin elevation due to Waldenström macroglobulinemia. For the other 11 patients where JEV-specific IgM was detected, IF for MVEV and WNV_KUNV_-specific IgM were also performed and found to be negative; none had a suggestive travel history requiring testing for other flaviviruses.

In eight of these eleven patients, a JEV-specific IgG titre rise of ≥4-fold was also observed, consistent with the diagnosis of acute JE (Table 3). Overall, JEV-specific IgG was detected in 84 samples (12.0%) from 75 patients (11.3%). Detailed clinical, exposure and vaccination histories were beyond the scope of the current study, hence the attribution of these remaining positive IgG results was not assessed in detail. Given the low JEV vaccination rates in the Australian population (administered only to travellers to endemic regions prior to 2022) and the known circulation of MVEV and WNV_KUNV_ in rural regions of NSW, the detection of IgG most likely reflects historical, non-JEV flavivirus exposure. Indeed, prior NSW data suggest a seroprevalence for flaviviruses of approximately 9% [22], though some geographic variability is expected.

JEV-specific IgM was detected in CSF in a single case, which had also tested positive for JEV-specific IgM in serum, consistent with the diagnosis of JE (Table 3). In a further 144 CSF samples from 139 patients, JEV-specific IgM was not detected.

Ten post-mortem brain tissue samples from one patient in the early phase of the outbreak underwent mNGS testing. Pooled samples from the left hippocampus, left midbrain, pons, medulla, right cerebellum and dentate nucleus identified sequences that aligned with published JEV genotype IV sequences, which was confirmed subsequently by JEV-specific RT-PCR testing. Further, deep RNA sequencing of individual samples recovered variable genome coverage (13.1%–98.9%), with the near-complete genome recovered from the left hippocampus tissue.

RT-PCR testing was performed on 114 CSF, 13 brain tissue, 40 blood and 49 urine samples collected for prospective testing. Of these, JEV RNA was detected in 11 samples from two patients, confirming the diagnosis of JEV infection. JEV-specific antibodies were not detected in one of these cases but were detected in the other aforementioned case that was diagnosed using mNGS (Case 1, Table 3). The case detection rate by RT-PCR was highest for NAAT of brain tissue (25.0%) and lowest in urine and blood (both 0%); the RT-PCR positivity rate from CSF was low (0.9%) (Table 2).

**Figure 2:**
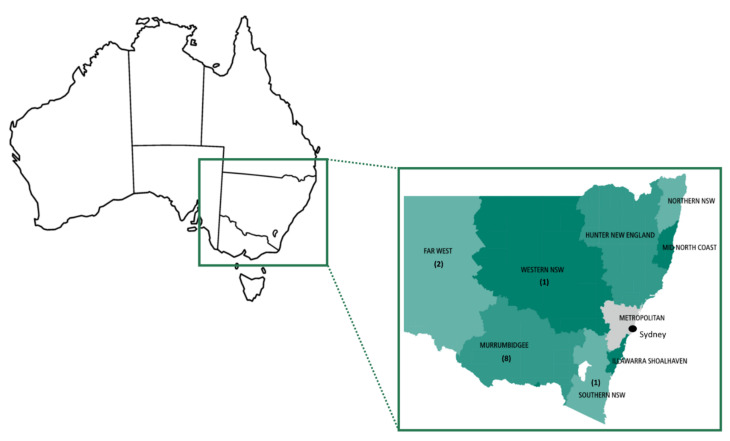
Location of confirmed cases of Japanese encephalitis in New South Wales by local health district. Number of cases for each local health district indicated in parentheses.

Overall, 12 cases of JE were confirmed in our laboratory over the first three months of the outbreak in NSW, representing a case detection rate of 1.4%. One case was diagnosed by both NAAT (mNGS and RT-PCR) and serology, one case by RT-PCR of CSF alone and ten cases by serology alone. The median age of patients from NSW diagnosed with JEV was 49.7 years (interquartile range 30.4 to 65.9 years, range 10.1 to 73.7 years), with a male:female ratio of 2:1. A single paediatric case, a 10-year-old child, was diagnosed in NSW during this time. Overall, the incidence of JE in NSW was 0.15 cases per 100,000 population over the study period. Positive cases resided in regions across Western NSW from the Victorian border in the south to the Dubbo region in the north (Figure 2).

No historical cases of JE were identified on retrospective JEV-specific IgM and/or RT-PCR testing of 145 stored CSF samples collected from 127 patients with clinical encephalitis but without a defined cause, in the six months preceding the first NSW case of JEV in the 2022 outbreak.

## 4. Discussion

The current unexpected JEV outbreak in Australia is of significant public health concern given the high morbidity and mortality of encephalitis caused by this virus, particularly in a highly susceptible population where the virus is newly emergent and non-endemic. As at 18 August 2022, a total of 40 human cases of JEV (30 confirmed and 10 probable) had been detected in Australia as part of the current outbreak [23].

Our data confirm that serology remains the cornerstone for JEV human case detection, with 11 of 12 cases in our cross-sectional analysis detected by this method with or without concurrent NAAT confirmation. Although the diagnostic sensitivity of serology is greater than that of NAAT given the short-lived and low level viraemia in humans, it is less specific due to the potential for cross-reactivity from shared epitopes across closely related flaviviruses. Over-interpretation of single acute antibody titres or a lack of appreciation of this possible cross-reaction by those less familiar with the nuances of flavivirus diagnostics can lead to misdiagnoses of JEV, particularly in regions where multiple flaviviruses are co-circulating. Similarly, haematological disorders such as plasma cell dyscrasias or Waldenström macroglobulinemia, associated with overproduction of immunoglobulins, may provide non-specific antibody titre elevations. Commercial assays [24,25], though recommended by the World Health Organization [26], are prone to the same issues of cross-reactivity. Alongside the technical expertise required for assay development and implementation, centralisation of testing in reference laboratories enables case-by-case interpretation and bespoke reporting of JEV testing results, given the high degree of background flavivirus seroprevalence known from prior studies [22] and inferred from our data. This is also important to accurately delineate the extent of the current, and future, JEV outbreaks.

NAAT, though limited by low and transient levels of virus in blood and CSF, provides high diagnostic specificity in patients sampled early in their illness. The kinetics of JEV viraemia are not well described in humans, though in other mammals such as pigs and hamsters, peak JEV RNA levels occur in blood from day three to five of illness [27,28]. Although described, the detection of JEV RNA in blood and urine, extending to day 26–28 [29], is rare. The overall case detection rate of 1.4% by RT-PCR in our cross-sectional analysis suggests low JE detection rates if inferred by NAAT alone. Blood and urine were particularly low-yield in our analysis, with no cases detected using these specimen types, though sample numbers were low. Conversely, targeted NAAT on brain biopsy specimens in the context of a suggestive but not necessarily specific clinical history of JE enabled an accurate diagnosis in one key sentinel case. The extrapolation of this finding is limited by our very small sample size for tissue specimens. In the context of an encephalitic illness of sufficient severity to warrant brain biopsy, a higher viral load in brain tissue compared to other sample types is plausible but remains speculative until a more extensive data set is available to confirm or refute this hypothesis.

In the current outbreak, mNGS was a key tool in recognising unsuspected JEV infection due to the emergent nature of the outbreak. Timely diagnosis of the first case of JE in the current outbreak in NSW (Case 1, Table 3), which occurred prior to establishment and validation of our targeted RT-PCR assays, was enabled using this powerful methodology. mNGS can be an invaluable tool to diagnose infectious causes of encephalitis, particularly in cases where a pathogen has not been detected by available targeted RT-PCR assays [30]. Pathogen-agnostic testing approaches such as mNGS enable unexpected pathogens to be uncovered, greatly enhancing existing diagnostic frameworks [31]. Cost, accessibility and the requirement for detailed bioinformatic expertise remain major obstacles for mNGS use as part of routine diagnostics. Nonetheless, clinical demand should ensure that mNGS is more available for challenging and complex cases of encephalitis, provided its limitations as a routine diagnostic method can be overcome [32].

In the current setting in Australia, testing for JEV is presently focused on patients with unexplained encephalitis (clinical signs and/or CSF pleocytosis and negative testing for more common pathogens). Such patients who reside in or have travelled to, or near, areas where JEV has been detected are considered at increased risk for JE given the role of pig or wild bird populations as amplifying hosts and reservoirs for the virus, respectively. However, it is recommended that clinicians refer to the most up-to-date JEV guidance regarding testing recommendations given the rapidity of updates in the current outbreak environment.

This cross-sectional study of JEV infection in an emergent setting has several limitations. Some testing modalities reported in this study, such as mNGS and RT-PCR on brain tissue, were performed on small numbers of samples, hence the conclusions herein must be interpreted with caution. Furthermore, diagnostic resources were necessarily concentrated on clinical cases of undiagnosed encephalitis due to clinical urgency during the initial phases of the outbreak; hence, overall prevalence is likely understated and certainly does not encompass non-encephalitic or asymptomatic JEV infections. As this study was focused on the laboratory aspects of diagnosis, clinical data were not available to provide further resolution for case detections. Ascertaining detailed clinical and epidemiological features of locally acquired JE cases, particularly if correlated with laboratory findings, will be highly informative to understand the utility of the various testing modalities in the context of different JEV-related symptom complexes (for example, comparing encephalitic versus non-encephalitic disease or assessing sensitivity of RT-PCR in various sample types, stratified by day from symptom onset). Furthermore, for the series of confirmed JEV cases in this study, the diagnostic yield of the different modalities could not be accurately compared, largely due to a lack of availability of CSF, blood and urine samples. While there may be several clinical reasons for the omission of CSF sampling, a large dataset of prospective cases with appropriate education of clinicians regarding sampling of acute and convalescent sera, as well as CSF, blood and urine early in illness, should enable more definitive comparisons between performance of the diagnostic modalities for JEV. Larger datasets encompassing both clinical and laboratory information are awaited to inform this important analysis.

The prevalence of JE remains low in NSW and is largely confined to inland rural and regional areas across the south and west of the State, though the current analysis is limited by sample numbers and sampling bias. As encephalitis represents the tip of the iceberg of JEV infections, broader ongoing surveillance across the geographical and clinical spectra will be critical to elucidate the extent of the JEV outbreak in Australia.

A broad understanding of JEV seroprevalence and the incidence of non-encephalitic disease in Australia is still lacking. Prospective and iterative serosurveys in humans are critical to understanding the prevalence and distribution of JEV infection in Australia in the coming years, and these should encompass both targeted testing of “high-risk” regions (such as amongst piggery workers or in geographically confined locations adjoining affected piggeries) as well as in the Australian population-at-large. The inclusion of participants across the age ranges will help to further delineate at-risk groups such as children or the elderly and elucidate differential exposure and serological response profiles by age or risk group.

Additional surveillance across mosquito, bird, pig and other non-human populations must include the sampling of similar “high-risk” areas as well as representative locations across the state. This animal and environmental surveillance data will play an equally important role in ascertaining the full ecological framework of the JEV life cycle in Australia and in planning a concerted OneHealth response to this JEV outbreak that encompasses the needs of all stakeholders.

Due to the geographical extent across which clinical cases and infected swine have been detected, and the number of infected piggeries involved (more than 70 at the time of writing) [33], it is likely that JEV will become endemic in Australia, necessitating appropriate and ongoing surveillance and mitigation strategies across human, animal and environmental health sectors. The exact date and mechanism of JEV introduction into Australia remains cryptic. Throughout 2020 and 2021, an unprecedented concentration of medical and public health resources on combatting the Coronavirus Disease 2019 (COVID-19) pandemic may have detracted from broader surveillance efforts and contributed to delayed recognition of this virus. Its detection in early 2022 sent reverberations through the medical, agricultural and environmental sectors as an emergent pathogen in its own right and as a warning for other potential outbreaks to occur at the human–animal interface and the need for close inter- and intra-agency collaboration.

Existing diagnostic capacity must be reinforced to ensure systems are sufficiently robust to handle prolonged or recurrent outbreaks, to accommodate increased testing numbers should JEV become endemic and to explore the spectrum of JEV-related disease. In doing so, such robust diagnostic frameworks will facilitate high-resolution analysis on the extent and clinical impact of this emergent outbreak in the Australian population. Consequently, policymakers must prioritise dedicated funding streams in the medium-term to manage the anticipated surge in JEV testing numbers and, across the long-term, to enable surveillance for and early detection of other emergent pathogens. The 2022 calendar year has provided a case-in-point on the frequency with which pathogens may emerge and spread and the importance of building laboratory capacity to rapidly establish diagnostic frameworks that can inform the effective management of outbreaks of nascent pathogens or of known pathogens emergent in new geographic regions.

## 5. Conclusions

Utilising a multi-modal approach, encompassing serology and NAAT, a JEV diagnostic framework was established within a centralised reference laboratory in the context of an emergent genotype IV JEV outbreak in NSW, Australia. A JE incidence of 0.15/100,000 persons was noted in the first three months of the outbreak, though overall JEV infection rates (encompassing encephalitic and non-encephalitic disease) are almost certainly much higher. Targeted JEV testing alongside serosurveillance studies will be critical, particularly over the next summer season, to inform ongoing epidemiological trends and determine whether endemicity of JEV has been established in Australia. Notwithstanding the impact of this outbreak, diagnostic networks in Australia are well constructed to enable a OneHealth approach to emergent pathogens encompassing human, animal and environmental priorities. Such cross-disciplinary cooperation should be prioritised to ensure timely and efficient outbreak management. Emergent pathogens must remain front-of-mind for public health networks, laboratories and policymakers despite contemporaneous demands such as those presented by the COVID-19 pandemic.

## Figures and Tables

**Table 1:** Case definition for Japanese encephalitis based on national case definitions, and accepted sample types for Japanese encephalitis virus testing in this study.

Case Definition	Accepted Sample Types				
Laboratory Criteria	Serum	Cerebro-Spinal Fluid	Brain Tissue	Whole Blood	Urine
Isolation of JEV by viral culture; * or	-	-	-	-	-
Detection of JEV RNA; or	-	✓	✓	✓	✓
IgG seroconversion or a significant increase in antibody level or a fourfold or greater rise in titre of JEV-specific IgG proven by neutralisation or another specific test, with no history of recent JE vaccination; or	✓	-	-	-	-
Detection of JEV-specific IgM in CSF, in the absence of IgM to MVEV, WNV_KUNV_ and dengue viruses; or	-	✓	-	-	-
Detection of JEV-specific IgM in serum in the absence of IgM to MVEV, WNV_KUNV_ and dengue viruses, with no history of recent JEV vaccination	✓	-	-	-	-

* Given short-lived and low level viraemia of JEV in humans, viral culture was not used as a diagnostic tool for acute JE in our laboratory. CSF, cerebrospinal fluid; JEV, Japanese encephalitis virus; MVEV, Murray Valley encephalitis virus; RNA, ribonucleic acid; WNV_KUNV_, West Nile virus (Kunjin variant).

**Table 2:** Japanese encephalitis testing summary for persons from New South Wales, Australia, including all prospective samples tested over the period 4 March 2022 to 31 May 2022.

		Positive (“Detected”) Samples, n (%)	Invalid/Equivocal Results, n	Negative (“Not Detected”) Samples, n	Number Samples Tested	Number Participants Tested	Case Detections, n (%)
**Serology**							
	**All**	90 (10.7%)	1	754	845	766	79 (10.3%)
	**CSF JEV IgM IF**	1 (0.7%)	-	144	145	140	1 (0.7%)
	Serum JEV IgM IF *	13 (1.9%) ‡	1	686	700	663	11 (1.7%)
	**Serum JEV IgG IF †**	84 (12.0%)	-	616	700	663	75 (11.3%)
**RT-PCR**							
	**All**	11 (5.1%)	7	198	216	145	2 (1.4%)
	**CSF**	1 (0.9%)	6	107	114	112	1 (0.9%)
	**Brain tissue**	10 (76.9%)	1	2	13	4	1 (25.0%)
	**Blood**	0 (0.0%)	0	40	40	37	0 (0.0%)
	**Urine**	0 (0.0%)	0	49	49	47	0 (0.0%)

* 2 samples (2 participants) referred but insufficient**;** † 1 sample (1 participant) referred but insufficient**;** ‡ Note: 11 of these 13 cases were deemed true positive cases for JE; one was due to cross-reacting antibodies from a recent Zika virus infection and one due to non-specific immunoglobulin elevation**;** CSF, cerebrospinal fluid; IF, immunofluorescence assay; JE, Japanese encephalitis; JEV, Japanese encephalitis virus; RT-PCR, real-time polymerase chain reaction.

**Table 3:** Diagnostic results for the twelve confirmed cases of Japanese encephalitis from New South Wales tested in our laboratory over the period 4 March 2022 to 31 May 2022.

Case #	CSF JEV IgM IF *	Serum JEV IgM IF *	Serum JEV IgG (Interval)	JEV RT-PCR	mNGS	Confirmed/Probable JE
1	detected	detected	>8× titre rise (7 days)	detected (brain tissue and CSF)	JEV sequence detected (brain tissue)	confirmed
2	not detected	not detected	not detected	detected (CSF)	-	confirmed
3	not detected	detected	4× titre rise (29 days)	not detected (CSF)	-	confirmed
4	-	detected	>4× titre rise (7 days)	-	-	confirmed
5	-	detected	falling IgG titres	-	-	confirmed
6	-	detected	4× titre rise (36 days)	-	-	confirmed
7	-	detected	>16× titre rise (9 days)	-	-	confirmed
8	-	detected	>4× titre rise (25 days)	-	-	confirmed
9	-	detected	>4× titre rise (19 days)	-	-	confirmed
10	-	detected	detected; no convalescent serum available	-	-	confirmed
11	-	detected	detected; no convalescent serum available	-	-	confirmed
12	-	detected	>8× titre rise (41 days)	-	-	confirmed

CSF, cerebrospinal fluid; IF, immunofluorescence; JE, Japanese encephalitis; JEV, Japanese encephalitis virus; mNGS, metagenomic next generation sequencing; RT-PCR, real-time polymerase chain reaction**;** * for all samples in which JEV IgM was detected by IF, MVEV and WNVKUNV IgM (and dengue, yellow fever and/or Zika IgM if appropriate travel history) was performed by IF and found to be negative.

## Data Availability

Not applicable.

## Abbreviations

CDINS	Communicable Disease Incident of National Significance
DEB	defined epitope blocking
ELISA	enzyme-linked immunosorbent assay
IF	immunofluorescence
IQR	interquartile range
JE	Japanese encephalitis
JEV	Japanese encephalitis virus
mNGS	metagenomic next generation sequencing
MVEV	Murray Valley Encephalitis Virus
NAAT	nucleic acid amplification testing
NSW	New South Wales (Australia)
RT-PCR	real-time polymerase chain reaction
SARS-CoV-2	severe acute respiratory syndrome coronavirus-2
WNV	West Nile Virus
WNV_KUNV_	Kunjin variant of West Nile Virus
